# Phase Field Study of the Microstructural Dynamic Evolution and Mechanical Response of NiTi Shape Memory Alloy under Mechanical Loading

**DOI:** 10.3390/ma14010183

**Published:** 2021-01-02

**Authors:** Shangbin Xi, Yu Su

**Affiliations:** Department of Mechanics, School of Aerospace Engineering, Beijing Institute of Technology, Beijing 100081, China; shangbinxi@bit.edu.cn

**Keywords:** shape memory alloy, super-elasticity, phase field, martensite multi-variants

## Abstract

For the purpose of investigating the microstructural evolution and the mechanical response under applied loads, a new phase field model based on the Ginzburg-Landau theory is developed by designing a free energy function with six potential wells that represent six martensite variants. Two-dimensional phase field simulations show that, in the process of a shape memory effect induced by temperature-stress, the reduction-disappearance of cubic austenite phase and nucleation-growth of monoclinic martensite multi-variants result in a poly-twined martensitic microstructure. The microstructure of martensitic de-twinning consists of different martensite multi-variants in the tension and compression, which reveals the microstructural asymmetry of nickel-titanium (NiTi) alloy in the tension and compression. Furthermore, in the process of super-elasticity induced by tensile or compressive stress, all martensite variants nucleate and expand as the applied stress gradually increases from zero. Whereas, when the applied stress reaches critical stress, only the martensite variants of applied stress-accommodating continue to expand and others fade gradually. Moreover, the twinned martensite microstructures formed in the tension and compression contain different martensite multi-variants. The study of the microstructural dynamic evolution in the phase transformation can provide a significant reference in improving properties of shape memory alloys that researchers have been exploring in recent years.

## 1. Introduction

The majority of advanced materials have the characteristics of a multi-phase or multi-domain structure [1], and the diffusion-less solid-solid transformation between these phases or domains results in some excellent thermal and mechanical properties, such as shape memory effect (SME) and super-elasticity [2,3,4]. Based on these unique properties and as one of the most extensive applications of shape memory alloys (SMAs), NiTi SMA has been widely used to develop intelligent system drivers and advanced devices [5,6,7,8,9]. The SME and super-elasticity of NiTi SMA are dependent on the multi-phase and multivariant martensitic microstructures [10,11]. The transformation of heterogeneous microstructures takes place if a thermal and/or mechanical load is applied, and the thermo-mechanical microscopic mechanism of the transition can be used to improve and optimize the properties of NiTi SMA.

Martensitic phase transformation or inverse transformation occurs in NiTi SMA by temperature-induced or stress-induced values, which are reasons why the NiTi SMA has SME and super-elasticity [4,12,13,14]. Isothermal loading and unloading below the martensite finish temperature (M_f_) induce the martensitic detwinning, requiring heat absorption to revert to a B2 phase and eliminate the residual strain, which shows SME on the macro scale [15,16,17]. Isothermal loading and unloading above the finish temperature (A_f_) of the B2 phase induce martensitic phase transition and reverse phase transition, fully recovering the accumulated deformation in the phase transition, which shows super-elasticity behavior on the macro scale [18,19,20]. During the transition from the cubic B2 phase (high-temperature phase) to the monoclinic B19′ phase (low-temperature phase), there are 12 B19′ variants that appeared in three-dimension space. According to the symmetry of the stress-free transformation strain of martensite variants, there are six B19′ variants in total in the two-dimensional plane. However, these phases/B19′-variants, i.e., nucleation—growth—degeneration—disappearance, are shifting rapidly in the phase transformation. Hence, it is difficult currently to track the multivariant microstructural evolution in real time with experimental methods.

In recent years, the phase field method based on the Ginzburg-Landau theory has been widely applied to study the evolution of the domain structure of materials [21,22,23,24,25]. The phase field method can better simulate the complexity and nonlinearity of phase evolution of SMAs during the phase transformation [25,26,27,28,29]. New phase field models have been proposed continuously to study the SME and super-elasticity of NiTi SMA. Ke et al. [22] developed a three-dimensional phase field model and studied the twinning interfaces formed between different pairs of B19′ variants. The simulation results show that the martensite variants are self-accommodated in the B2–B19′ transformation and attain the twin patterning with only two B19′ variants. Mamivand et al. [30] established a two-dimensional phase field model of elastic heterogeneous tetragonal to monoclinic phase transition, and simulated the orientation relationship and symmetry reduction between the parent phase and generative phase during the transformation of Zirconia at a constant temperature. The results show that the different boundary conditions lead to a completely different twinning pattern and phase volume fraction. Zhong et al. [23] developed a phase field model with twelve B19′ variants and studied the B2 to B19′ martensitic phase transformation of NiTi SMA. The results show the formation of poly-twined martensitic microstructure and the factors influencing the pattern of martensitic twin variants, such as the mechanical constraints and crystallographic orientation. By improving the phase field models of Levitas et al. [31] and Idesman et al. [32], Esfahani et al. [25] established a phase field model, which is scaling independent from a cubic phase to a monocline phase transformation to study the influence of the strain rate and crystal orientation on stress-strain response and microstructure evolution. The results show that the effects of the external strain rate on the microstructure and overall stress-strain response are very small. Li and Su [33] established an isothermal phase field model for SMA. They systematically studied the self-accommodated nucleation and growth of the martensite variants at low temperature (i.e., below the martensite finish temperature) and the detwinning process with the strain-rate effect. The results show that the intense evolution of the microstructure results in a stress plateau in the loading. Xu et al. [34] studied the one-way SME of NiTi SMA by the developed phase field method, and demonstrated that a martensite variant has the lowest elastic energy and enjoys the greatest growth advantage when it grows along with the interface. To some extent, it reflects the evolution of the microstructures in the one-way SME of NiTi SMA.

Although the phase field researches of SMAs mentioned above, including the Refs. [35,36,37,38], involve mechanical properties and the evolution of the phase structures, either the most phase field simulations of NiTi SMA study the microstructural evolution in a stress-free state, or the phase field models contain only two martensite variants because it is very difficult to construct a local free energy function containing twelve energy potential wells and attain the theoretical solution of martensite multi-variants compatibility at a twin boundary [23]. It is very difficult to obtain the multivariant martensitic (i.e., four or more) twinned pattern. Moreover, there are few research studies that probe the conversion between martensite variants in the SME and super-elasticity of NiTi SMA, which have an extremely important impact on the mechanical properties of NiTi SMA. Thus, a phase field model that can describe the multi-variants microstructure continuous response under applied loading of NiTi SMA is our desideratum.

In this paper, we employed a new energy-barrier expression for temperature below the equilibrium temperature T_0_ to yield a metastable austenite phase below the martensite finish temperature M_f_. Therefore, a new phase-field model was developed for a non-isothermal process wherein the effect of varying ambient temperature on the martensitic transformation can be investigated. In this model, the local free energy function possesses six energy potential wells for the entire six martensitic variants in two dimensions. With the phase-field model, we investigated the microstructural evolution of NiTi SMA during the complete cycle of the shape memory effect, namely, the thermally induced martensite twinning, the stress-induced martensitic detwinning, the unloading process, and the thermally induced martensite-to-austenite transformation. In the meantime, we investigated the correlation between the microstructure evolution and the overall stress-strain behavior for the super-elastic deformation under an applied mechanical load. The simulated results reveal the microscopic mechanism of a mechanical response of NiTi SMA under external load and provide a reference for improving the mechanical properties of SMA.

## 2. Phase Field Model

The extraordinary thermodynamic properties of SMAs are derived from the martensitic phase transformation. The decrease or increase in temperature can induce forward or reverse martensite transformation of NiTi SMA, and mechanical action (stress or strain load) can produce the same effect. The phase field method is performed to simulate the multivariant martensitic microstructure through a set of continuum order parameters $\varphi_{i}\left(=1,2,\ldots ,6\right)$ between zero and one, which are used as an indicator of the phase at each material point system in a phase field model. The phase field model provides the solutions of the temporal-spacial evolution by solving the time-dependent partial differential equations of these order parameters numerically.

### 2.1. Phase Field Equation

The microstructural evolution in phase transition of NiTi SMA is governed by the time-dependent Ginzburg-Landau equation, which is based on the idea that the free energy can be expanded as a power series in the order parameter φ. If $\varphi_{1}=\ldots =\varphi_{6}=0$, it indicates that the microstructure of NiTi SMA is an austenite B2 phase at this point. The phase field equation can be written as:

(1) $$\frac{\partial \varphi_{i}}{\partial t}={Κ}_{ij}\left(\frac{\partial \psi }{\partial \varphi_{i}}-\frac{\partial }{\partial x_{j}}\left(\frac{\partial \psi }{\partial \varphi_{i,j}}\right)\right),$$

where $\varphi_{i}$ represents the ith variant of martensite and ${Κ}_{ij}$ is the matrix of kinetic coefficients. For simplicity, ${Κ}_{ij}$ is set as the product of a constant k and a diagonal matrix $\delta_{ij}$, and k is set as 1.25 J/m^3^s. ψ denotes the total energy density of the system.

### 2.2. Local Free Energy

Microstructural evolution of a material takes place to decrease the total free energy $\Psi_{t}$ of the system, which can represent transformation involving the reduction of symmetry between the parent phase and produced phase containing martensite multi-variants, and it is usually expressed as the volume integral of the free energy density ψ, and can be written as:

(2) $$\Psi_{t}=\int_{V}\psi dV$$

In the martensitic phase transformation of NiTi SMA, the total free energy density ψ of system is the summation of local energy density $\psi_{local}$, gradient energy density $\psi_{grad}$, and elastic strain energy density $\psi_{el}$, i.e.,

(3) $$\psi =\psi_{local}+\psi_{grad}+\psi_{el}\;.$$

The local energy density $\psi_{local}$ is temperature-dependent and the fourth-order Landau-type polynomial in the stress-free state depends on the thermodynamic properties of material. It can be described as:

(4) $$\psi_{local}=\frac{A_{1}}{2}\sum_{i}^{n}\varphi_{i}{}^{2}-\frac{A_{2}}{3}\sum_{i}^{n}\varphi_{i}{}^{3}+\frac{A_{3}}{4}{\left(\sum_{i}^{n}\varphi_{i}{}^{2}\right)}^{2}\;,$$

where the Landau-type coefficients $A_{1}$, $A_{2}$, and $A_{3}$ are the temperature-dependent and must satisfy the constraint conditions, i.e.,

(5) $$\{\begin{array}{l} \psi_{local}^{\prime }\;(1,T)=A_{1}-A_{2}+A_{3}=0\\ \psi_{local}^{\prime \prime }\;\left(1,T\right)=A_{1}-2A_{2}+3A_{3}>0.\\ \psi_{local}^{\prime \prime }\;\left(0,T\right)=A_{1}>0 \end{array}$$

Thus, the martensite and austenite phase are steady state. If the Gibbs energy barrier $\Delta G^{*}$ is given, the Landau coefficients can be expressed as $A_{1}=32\Delta G^{*}$, $A_{2}=3A_{1}-12\Delta G$, and $A_{3}=2A_{1}-12\Delta G$ [39]. According to the previous research studies [38,40], the energy barrier of martensitic phase transformation is independent of temperature below equilibrium temperature $T_{0}$. In order to make the austenite phase metastable of NiTi single crystal below an equilibrium temperature, we take as:

(6) $$\Delta G^{*}=0.2Q/32\;(when\;T\leq T_{0}).$$

when $T>T_{0}$, the energy barrier is positively correlated to ambient temperature [41,42], and it is taken as $\Delta G^{*}=[0.8+0.06(T-T_{0})]Q/32$. ΔG denotes the difference of the chemical free energy density between the austenite and martensite phase, and it depends on temperature. In addition, it is also called a phase transformation driving force, which is the difference of the local free energy of cubic austenite and monoclinic martensite phase, and can be expressed as:

(7) $$\Delta G(T)=Q(T-T_{0})/T_{0}\;,$$

where Q is the phase transform latent heat and can be set as $Q=110\;\;MJm^{-3}$. $T_{0}$ is the equilibrium temperature and can be assumed as the following:

(8) $$T_{0}=(A_{s}+M_{s})/2\;,$$

where $A_{s}$ is the start temperature of austenite transformation and $M_{s}$ is the start temperature of martensite transformation. In our study, $M_{s}=334\;K,$ $M_{f}=313\;K,$ $A_{s}=341\;K$, and $A_{f}=358\;K$, referring to Xu et al. [43], so $T_{0}=337.5\;K$.

The elastic energy density, $\psi_{el}$, is a deformation energy stored in the phase transformation, and is given by the following.

(9) $$\psi_{el}=\frac{1}{2}C_{ijkl}^{A-M}\varepsilon_{ij}^{el}\varepsilon_{kl}^{el}\;,$$

where $\varepsilon_{ij}^{el}$ and $\varepsilon_{kl}^{el}$ are elastic strain tensors given by the stress-free transformation strain $\varepsilon_{ij}^{*}$ and the total strain $\varepsilon_{ij}$, i.e.,

(10) $$\varepsilon_{ij}^{el}=\varepsilon_{ij}-\varepsilon_{ij}^{*}.$$

The total strain $\varepsilon_{ij}$ can be defined by partial derivatives with respect to spatial coordinates $x_{i}$ and $x_{j}$, as follows.

(11) $$\varepsilon_{ij}=\frac{1}{2}\left(\frac{\partial u_{i}}{\partial x_{j}}+\frac{\partial u_{j}}{\partial x_{i}}\right)\;.$$

The constitution equation can be obtained by using the Hook’s law.

(12) $$\sigma_{ij}=C_{ijkl}^{}\varepsilon_{kl}^{el}\;.$$

where $C_{ijkl}$ denotes the elastic coefficient matrix in the transition of the B2-phase to B19′-phase. Additionally, during the martensitic phase transformation of NiTi SMA, the gradient energy density $\psi_{grad}$ can be written as:

(13) $$\psi_{grad}=\frac{1}{2}\sum_{p=1}^{n}\beta_{ij}(p)\frac{\partial \varphi_{p}}{\partial x_{i}}\frac{\partial \varphi_{p}}{\partial y_{i}}\;,$$

where the coefficients $\beta_{ij}(p)$ are the components of a semi-positive defined gradient energy tensor, which depends on the direction of the gradient decided by spatial derivatives of the order parameters $\varphi_{p}$ with respect to coordinates $x_{i}$ and $x_{j}$. In this paper, we assume that gradient energy coefficient tensors are isotropic for simplicity, that is, $\beta_{ij}=\beta \delta_{ij}$, and we set β=1.0 [33].

### 2.3. Mechanical Equilibrium Equation and Boundary Condition

Both the stress and strain tensors are second-order tensors represented by $\sigma_{ij}$ and $\varepsilon_{ij}$, respectively, in the Cartesian coordinate system. The mechanical equilibrium equations are given with the usual forms.

(14) $$\sigma_{ji,j}+b_{i}=\rho {\ddot{u}}_{i}\;(\mathrm{in}\;V),$$

where $b_{i}$ is the external body force and $\rho {\ddot{u}}_{i}$ is the inertial term. In this work, we set both the external body force and inertial term as zero. Thereby, the evolutions of order parameters are traced in real time by solving the mechanical equilibrium equations and Ginzburg-Landau equations simultaneously.

The boundary conditions are given by:

(15) $$\sigma_{ji}n_{i}\;-\;t_{j}=0\;(\mathrm{on stress boundary}\;\Gamma )$$

where $n_{i}$ is the direction cosine of an outward pointing normal to the boundary and $t_{j}$ is the surface force. In order to trace the evolution of each order parameter, we integrate all of the governing equations.

### 2.4. Model Parameters

We research six continuous field variables $\left\{\varphi_{1},\;\;\;\varphi_{2},\;\;\;\varphi_{3},\;\;\;\varphi_{4},\;\;\;\varphi_{5},\;\;\;\varphi_{6}\right\}$ to describe the different B19′ variants during phase transformation of NiTi SMA, which is sufficient to describe the nucleation and growth of the microstructure during the phase transition in 2D simulation. In addition, the six free-stress strain of $\varphi_{i}\;\left(i=1,2,\ldots ,6\right)$ in the phase transition are given by:

$$\varepsilon_{1}^{*}=\left(\begin{array}{cc} \alpha & w\\ w & \beta \end{array}\right),\;\varepsilon_{2}^{*}=\left(\begin{array}{cc} \alpha & -w\\ -w & \beta \end{array}\right),\;\varepsilon_{3}^{*}=\left(\begin{array}{cc} \beta & w\\ w & \alpha \end{array}\right),\;\varepsilon_{4}^{*}=\left(\begin{array}{cc} \beta & -w\\ -w & \alpha \end{array}\right),\;\varepsilon_{5}^{*}=\left(\begin{array}{cc} \beta & k\\ k & \beta \end{array}\right),\;\varepsilon_{6}^{*}=(\begin{array}{cc} \beta & -k\\ -k & \beta \end{array}),$$

where the components of $\varepsilon_{i}^{*}$ are α=−0.0437, β=0.0243, w=−0.0427, and k=0.0580, respectively [2]. Then, we employ the elastic constant matrix calculated by Hatcher et al. [44] in this work, i.e., $c_{11}=183\;\mathrm{GPa}$, $c_{12}=146\;\mathrm{GPa}$, and $c_{44}=46\;\mathrm{GPa}$ as the material parameters of NiTi SMA.

## 3. Results and Discussion

In this work, we use the finite element method to solve the Ginzburg-Landau equation. A simulation system of 60 nm×60 nm is discretized into a two-dimensional plane strain finite element model containing 28,800 structured triangular elements. It should be noted that one simulation time corresponds to 50 ns in real time. If no special instructions, colors in pictures of simulated microstructure morphologies represent different martensite variant of NiTi SMA.

### 3.1. The Microstructural Evolution of NiTi Single Crystal under a Fixed Temperature

**Figure 1:**
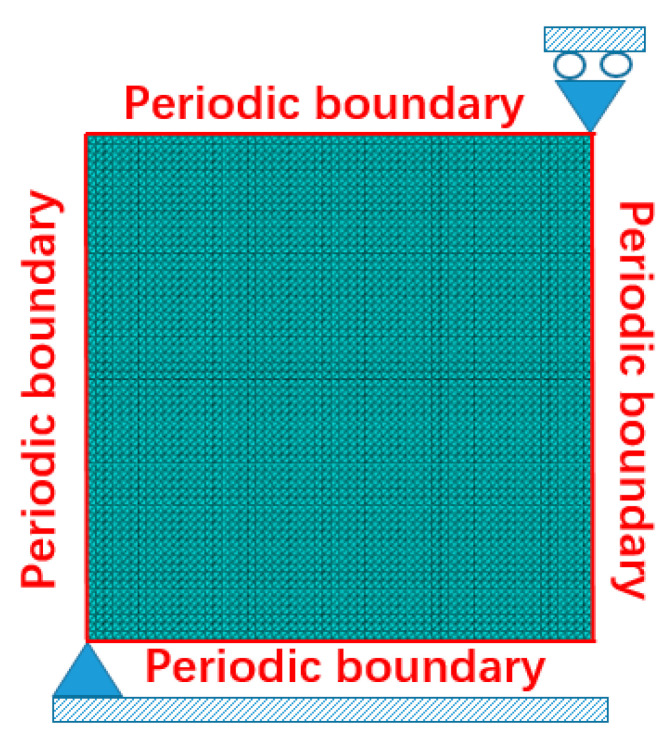
The periodic boundary conditions and mechanical constraints applied on the finite element model of the NiTi single crystal.

In this section, we simulate microstructural evolution of NiTi SMA at 310 K in the global Cartesian coordinate system. The periodic boundary conditions are applied to the four edges of the geometrical model to eliminate the boundary effect, and a constraint as shown in Figure 1 prevents the model from moving. We impose a set of random numbers between 0 and 1 of order parameters as the thermal fluctuations to promote the nucleation of martensite at the beginning of the simulation. Since our finite element model has good convergence of numerical integration, the total simulation time ${\tilde{t}}_{tot}=260$ is divided into 650 simulation steps.

**Figure 2:**
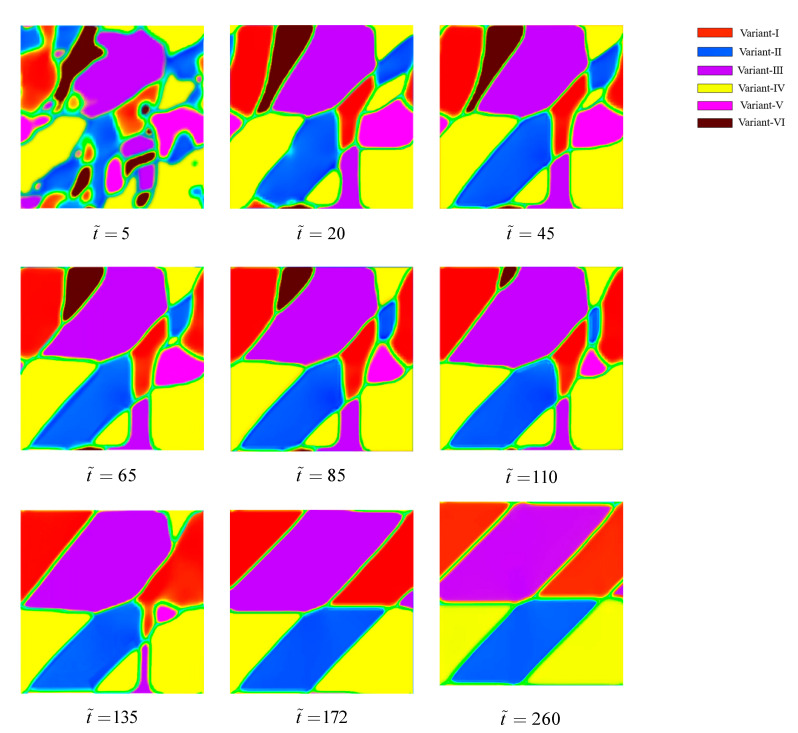
The microstructural morphologies at a different simulation time in the transformation from B2 to B19′, showing the formation of a poly-twinned martensitic microstructure.

Since the austenite phase is a metastable state at 310 K, the phase transformation of B2–B19′ occurs under the stimulation of thermal fluctuations. Figure 2 shows the nucleation and expansion of martensite variants, and a patterning of poly-twined martensitic microstructure is formed. The diving force of microstructural evolution is a combination of local free energy, elastic energy, and gradient energy that mutually lower the total energy of the system. At $\tilde{t}=5$, the martensite precursors microstructure emerges, which results in lattice distortion and the order parameters $\varphi_{i}$ deviate from all-zero values. The microstructure morphology is irregular and random distribution at this moment. At $\tilde{t}=20$, the nucleation of martensite variants is finished. It indicates that all order parameters $\varphi_{i}$ are near the vicinity of one, but no B19′ variants are stable completely. At this moment, the microstructure contains six martensite variants. With the evolution of the microstructure, the morphologies of a few B19′ variant domains structure become like-band at $\tilde{t}=45$. In order to balance the energy of the system, the finite element equations keep solving to obtain a “perfect” crystallographic solution that makes the energy of the system lower, resulting in the continuous changes in the microstructural morphology. In $\tilde{t}=45\text{\textasciitilde{}}110$, it is clear that the volume fractions of the variant-V and variant-VI in the microstructure are going down gradually. Soon after, the variant-VI disappears completely at $\tilde{t}=135$. The variant-V also disappears and poly-twinned patterning of martensite multi-variants is formed at $\tilde{t}=172$, while the interfaces of banded poly-twinned martensite are still not smooth. After a period of microstructural evolution, the poly-twinned martensitic microstructure is stable and twin boundaries are also smooth completely at $\tilde{t}=260$. The microstructural morphology of the material remains unchanged as the simulation time continues to increase. The pattern of poly-twinned martensitic structure is obtained by B19′ multi-variants that are self-accommodating. The patterns are similar to those obtained by Zhong et al. [23].

**Figure 3:**
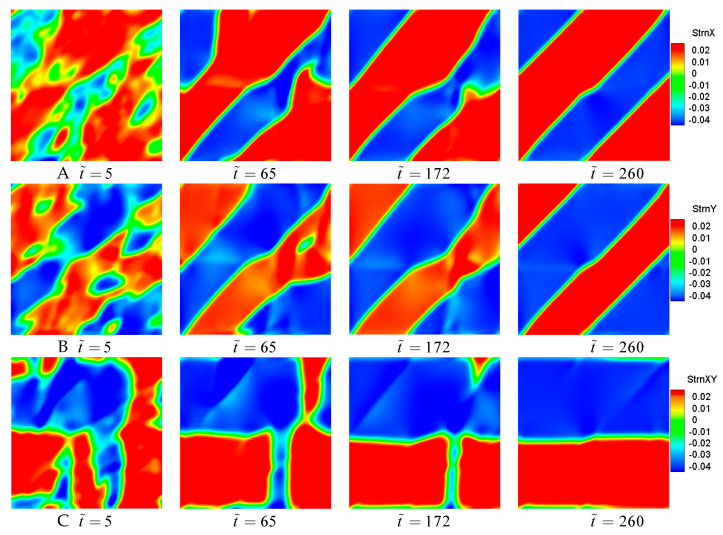
The evolution of logarithmic strain fields in the transformation from B2 to B19′ in the global Cartesian coordinate system. (**A**–**C**) show the evolution in the x-direction, y-direction, and xy-direction, respectively.

In order to explore the phase transformation of B2–B19′ further, the regional deformation in a different direction is calculated and shown in Figure 3. It can be seen that the evolution of the logarithmic strain field is similar to microstructural evolution. During the transformation from cubic B2 to monoclinic B19′ by the temperature induced, the nucleation and expansion of B19′ variants cause a local deformation resulting in changing the regional shape. The self-coordinated growth of the B19′ variant lowers the total elastic energy.

As seen from Figure 3, the local logarithmic strains in the x-direction, y-direction, and xy-direction are inhomogeneous at $\tilde{t}=5$. At $\tilde{t}=65$, the logarithmic strain fields become more homogeneous, and the local high strain reaches to −4% (“−” denotes the opposite direction of the axis). Although the pattern of poly-twinned martensitic microstructure forms at $\tilde{t}=172$ basically, the distribution of the logarithmic strain is not uniform. When the martensitic transformation finishes, the logarithmic strain field is uniformly distributed in the model, and the local deformation of the simulation region is obtained.

### 3.2. Microstructure and Mechanical Response Dependent on the SME

**Figure 4:**
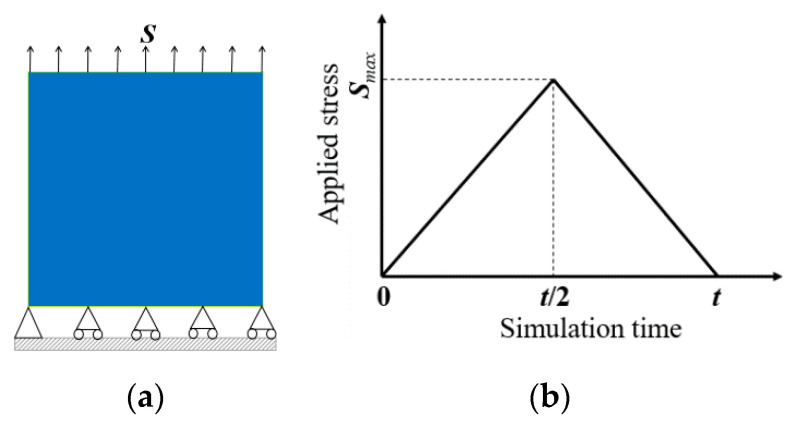
Mechanical boundary conditions impose on the finite element model, (**a**) stress loading and constraint of the model, and (**b**) time history of the uniaxial stress load.

In this section, the detwinning and SME of NiTi SMA are simulated to study the microstructural dynamic response under an applied load (stress/temperature load) and insight into the microscopic mechanism of material with excellent mechanical properties by the developed phase field model. First, the microstructure response under tensile and compressive stress load is studied at 310 K, and then the applied stress is released. Second, the finite element model is heated to above austenite finish temperature to investigate the SME of NiTi single crystal SMA. The horizontal freedom degree and vertical freedom degree of the node displacements at the left-bottom corner of the model (node-1 in a finite element model) are constrained, i.e., u1 = 0, u2 = 0, and other nodes at the bottom boundary of the geometric model constrain only the vertical displacement freedom degree, which avoids the motion of the geometric model as a stress load is applied, as shown in Figure 4a. In the loading stage, a stress load varied linearly from $\tilde{S}=0$ to a maximum value $\tilde{S}=S_{max}$ is imposed to the top boundary of the geometric model, and then the imposed stress load back to $\tilde{S}=0$, as shown in Figure 4b.

The poly-twinned martensitic microstructure is taken as the initial structure, and a stress load of 740 MPa is applied to the upper boundary of the model at 310K. The detwinning of the poly-twinned martensite take place in the process of loading since the B19′ variants possess a different stress-orientation.

**Figure 5:**
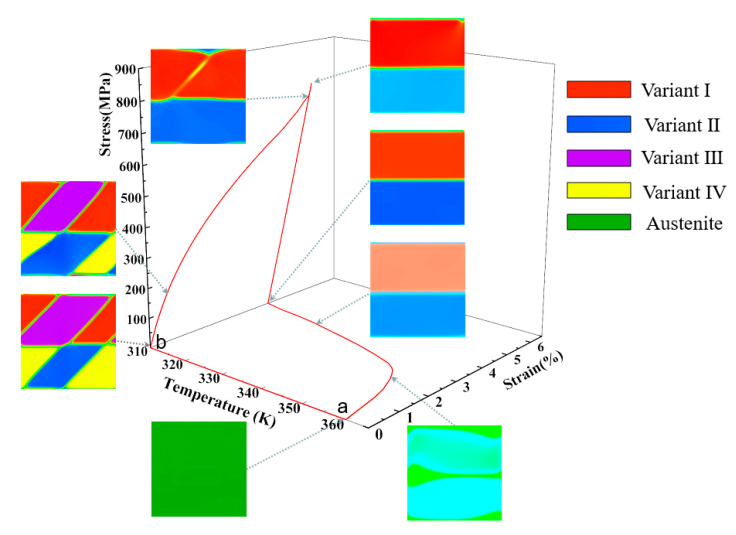
Stress-strain-temperature curve and microstructure evolution reflecting the SME of NiTi SMA single crystal under a tension stress.

Figure 5 shows the stress-strain-temperature curve and microstructural morphologies of the corresponding critical points on the curve obtained in the process of uniaxial tensile loading-unloading and heating. Note that the stress and strain are calculated by a mean method, i.e., the values of stress or strain of all elements in the single crystal are summed and then averaged by the area of the single crystal. At the initial loading stage, there is an extremely short period of approximately linear elasticity as the loading is done at a fixed temperature and without the process of the thermal homogenization. The order parameter $\varphi_{i}$ fluctuates exceedingly little, that is, the poly-twinned microstructure is almost constant. The detwinning of a twinned martensite structure takes place if the applied stress continues to increase to 175 MPa. The change of the microstructure manifests in that variant-I and variant-II annex variant-III and variant-IV gradually. The variant-III and variant-IV disappear completely when the applied stress reaches to 703 MPa, and then only the variant-I and variant-II microstructures remain. When the implemented stress continues to increase, the microstructure remains changeless and the stress-strain curve becomes nearly linear again. The applied stress is released immediately when it reaches $\sigma_{\max}$ (740 MPa), and the unloading rate is the same as the loading rate. The material microstructure morphology remain unchanged until the employed stress is released completely.

**Figure 6:**
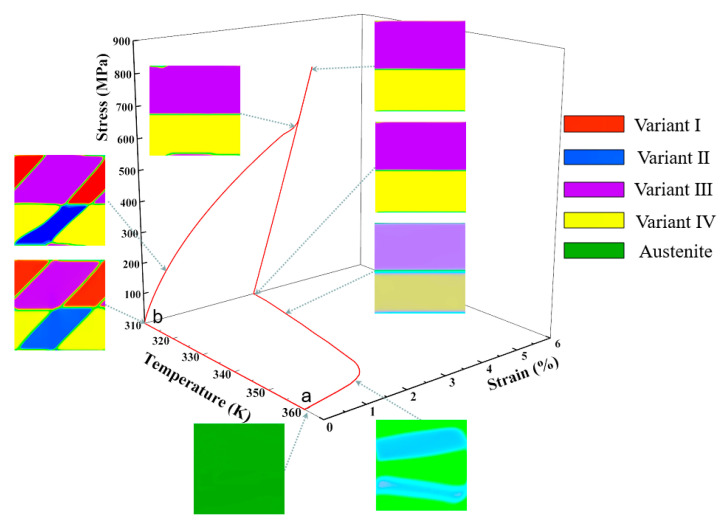
Stress-strain-temperature curve and microstructure evolution reflecting the SME of NiTi SMA single crystal under a compression stress.

In order to probe the SME of NiTi SMA, the temperature of the system is heated from 310 K to 360 K when the applied stress is unloaded to zero. The martensite reverse transformation takes place and the residual strain is eliminated due to increasing the total energy of the system. The results of numerical simulation demonstrate the SME of NiTi SMA, which shows that the martensite B19′ phase is transformed into austenite B2 phase on a micro level, and the deformation of the material is restored on a macro level. Segments a–b of Figure 5 and Figure 6 represent the formation of a poly-twinned martensite microstructure.

Figure 6 demonstrates the relationship of stress, strain, and temperature and the evolution of the poly-twinned microstructure in the uniaxial compression. The initial microstructure in the process of compression is the same as the simulation of tension. The loading rate during compression is the same as previous tension simulation, while the martensitic transformation during compression is more intense. In the early stage of compression loading, the domain structure share of variant-I and variant-II decrease rapidly, while variant-III and variant-IV increase continuously. When the compression load increases to 161 MPa, the domain structure shape and distribution of variants in the single crystal begin to change. The martensite phase transformation is completed when the compression load increases to 596 MPa. At this point, variant-I and-II disappear, and variant-III and variant-IV constitute the microstructure of the material, which is a result of the stress-accommodating of martensite variants. However, the interfaces of the variants’ domain structure are not flat, as shown in Figure 6. If the compression load continues to be increased, the NiTi SMA behaves approximately linearly elastic, which the microstructural interfaces become smoother and the order parameter values are closer to 1 at this stage. In addition, the microstructure of the material remains almost unchanged during unloading because the monoclinic martensite phase is more stable at this ambient temperature and there is no driving force for nucleating a new variant.

When the model is unloaded to a zero-stress state, we raise the temperature of the system gradually, which is the same as the tension simulation. An increase in temperature leads to an increase in local free energy with the rapid decrease in the values of order parameters. The transformation from martensite to austenite takes place, and the martensite B19′ variants recede gradually. The mode and size of temperature loading are the same as the tension simulation. The microstructure evolution of NiTi SMA in the compression is similar to the tension, while the response of temperature vs. strain is slightly different since a different variant corresponds to a different stress-free strain, which affects the total free energy density of the system.

It can be seen from the stress-temperature-strain curves that there are slight differences between the tension and compression simulation. The residual strain after unloading the tension is greater than the simulation of compression of the material, which is a result of stress-accommodation of variants. The critical stresses of detwinning finished are about 596 MPa and 703 MPa during compression and tension, respectively.

As can be acquired from the above research, the patterning of martensite multi-variants in the formation of stress-accommodating microstructures is determined by the mechanical loading. A different twinned structure is stabilized in the tension and compression, while each variant has the same geometrical morphology and volume fraction. In our phase field model, the numerical solutions that make the twin interfaces compatible can be obtained by applying accurate boundary conditions and resulting in the energy of the system reaching the minimum value.

### 3.3. Microstructure and Mechanical Response Dependent on the Superelasticity

Super-elasticity of SMA is an ability to fully recover large deformation caused by the applied load above an austenite finish temperature $A_{f}$. In order to investigate the microstructural evolution dependent on the super-elasticity behavior of the NiTi single crystal, the tension and compression tests are carried out at 360K. When the initial microstructure is the austenite B2 phase ($\varphi_{i}=0,\;i=1,2,\ldots ,6$), a stress load of 1.5 GPa is applied to the finite element model. The boundary conditions of the model are the same as the previous section exactly, as indicated in Figure 4a. The total simulation time $\tilde{t}=500$ in the process of loading-unloading simulation is divided into 1000 incremental steps.

**Figure 7:**
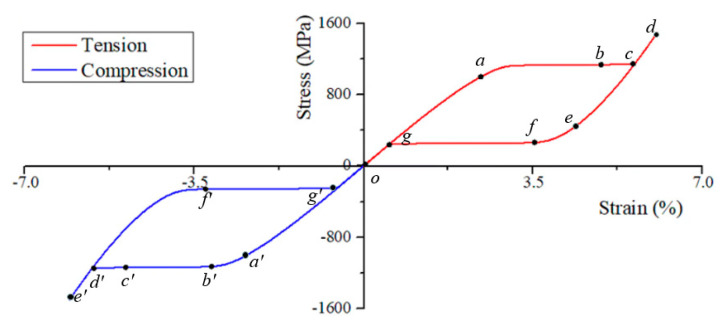
The stress-strain curves obtained from simulations of super-elasticity tension and compression test. The simulation time corresponding to the points on the curves are: on the tension curve, o: $\tilde{t}=0$ or $\tilde{t}=500$, a: $\tilde{t}=131$, b: $\tilde{t}=138$, c: $\tilde{t}=143$, d: $\tilde{t}=250$, e: $\tilde{t}=389$, f: $\tilde{t}=395$, g: $\tilde{t}=402$, respectively. On the compression curve, a′: $\tilde{t}=130$, b′: $\tilde{t}=133$, c′: $\tilde{t}=138$, d′: $\tilde{t}=147$, f′: $\tilde{t}=403$, respectively.

Figure 7 demonstrates the isothermal stress-strain response of NiTi SMA in the tension and compression case. It can be seen from the stress-strain curves that both tension and compression simulation have clear stress hysteresis in the unloading stage. The critical value of the phase transition is about 1.13 GPa in loading stages, which is slightly higher since the finite element model does not contain a crystal defect that promotes martensitic nucleation. The stain returns to zero after unloading. The results are consistent with the simulated results of Cui et al. [40].

**Figure 8:**
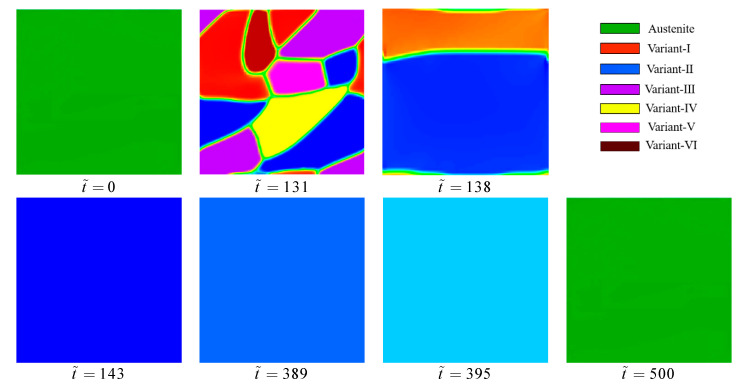
The 2-D phase-field simulation results in the tension of super-elasticity of the NiTi single crystal, showing the microstructural morphologies at a different simulation time.

Figure 8 manifests the formation and degradation of martensite variants during forward and inverse martensitic transformation. The initial state of the NiTi single crystal in stress-free configuration is austenite phase, and the microstructure is shown in Figure 8 (corresponding to point o in Figure 7). At the beginning of tension, the austenite phase deforms elastic and uniform values. At the simulation time $\tilde{t}=131$, the applied stress arrives at a certain critical value, and the martensitic phase transformation is triggered. Then, the martensite variants start to nucleate and grow, corresponding to point a in Figure 7. At this material point, the microstructure contains all martensite variants, while none of the variants reach a stable state. The morphologies of variants are irregular and random distribution. However, the subsequent variation of the microstructure is dramatic. The stage of transition takes only 24 incremental steps. It should be noted that there are 500 simulated incremental steps in the loading stage in total. At this stage, the martensite variants have a preferential orientation that the growth-friendly variants annex the growth-unfriendly variants gradually. It indicates that variant-III, variant-IV, variant-V, and variant-VI fade away while only variant-I and variant-II remain. We can see the color of variant-I becoming pale, which indicates that the value of $\varphi_{1}$ deviates from 1. When $\tilde{t}=143$, the variant-II disappears as well, and the microstructure of the material reaches a stable state. The result is consistent with that observed by Priyadarshini et al. [45] in an experiment that the band-type martensitic microstructure is formed in NiTi at a high temperature.

If the loading finishes, the applied stress is released as the same rate as the loading. The mechanical response of the material at the beginning of unloading is linear elasticity similarly to the initial stage of loading where the microstructure changes tardiness as well. The reverse martensitic transition takes place when the applied stress is unloaded to the critical value of 273 MPa. At $\tilde{t}=389$, $\varphi_{2}$ start to decrease, that is to say, the variant-II fades away, as shown in Figure 8. When the applied stress is unloaded to 261 MPa (point g in Figure 7), the transformation from the martensite to austenite phase is completed. In the unloading terminal, the values of order parameters $\varphi_{i}\left(i=1,2,\cdot \cdot \cdot ,6\right)$ continue to decrease slowly and become zero finally. At $\tilde{t}=500$, the applied stress is released to zero, and the microstructure of the material recovers to an austenite state. In the process of stress release, the reverse martensitic transition reduces the free energy of the system and recovers the deformation of material.

**Figure 9:**
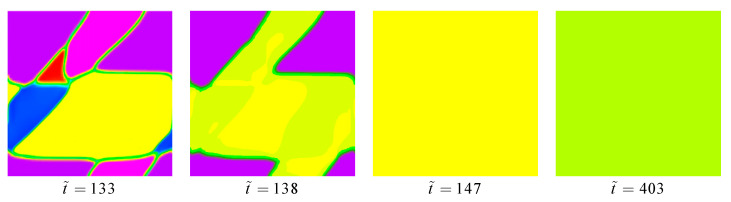
The 2-D phase-field compression simulation results of superelasticity, showing the evolution of multivariants microstructure of key points.

Figure 9 demonstrates the simulated microstructural evolution of the NiTi alloy under an in-plane vertical direction compressive stress of 1.5 GPa, and the rate of loading- unloading is the same as the tension simulation. The stress-strain responses in compression and tension tests are very similar. However, the formed patterning of the B19′ variant is stipulated by mechanical loading. Different from the tension simulation, variant-IV is more suitable for growing in the compression, which reflects the asymmetry of the microstructure between tension and compression. If the simulation time arrives to $\tilde{t}=130$, the stage of the elastic deformation of material ends, and the transformation from austenite to martensite starts. After only six incremental steps ($\tilde{t}=133$), the variant-VI disappears from the microstructure of the NiTi single crystal. When $\tilde{t}=138$, the variant-I, variant-II, and variant-V disappear as well since the stress-free transformation strains of these variants are challenged for stress-accommodation. When $\tilde{t}=147$, the microstructure of the NiTi single crystal becomes fully homogeneous, and only variant-IV remains under the applied stress. Sticking with stress-loading, the microstructure remains stable and the stress-strain curve passes into the linear as the martensitic phase transformation is finished, and the free energy of the system is close to the minimum. The martensitic microstructure remains constant until $\tilde{t}=403$, where the value of $\varphi_{4}$ starts to decrease as the applied stress is released. In the reverse martensitic transformation, the release of applied stress causes both local free energy and strain energy to decrease, resulting in the total energy of the system decrease.

Through the numerical study of super-elasticity behavior of NiTi SMA, the stress-strain response and microstructural evolution of NiTi SMA under tensile and compressive load are described. The simulated results are in good agreement with Janine et al. [46] and Anand et al. [47], studying in terms of the preferred orientation and stress-strain response of variants in the microstructure evolution. It indicates that the mechanical behaviors of NiTi SMA are dependent on martensite variants, and the mechanical loading determines the nucleation and growth of martensite variants.

## 4. Conclusions

In this work, a new phase field model is developed to study the microstructural evolution and mechanical response of NiTi SMA in phase transformation by the temperature-stress induction. We construct a Landau-type local free energy function with six martensite variants to describe the microstructural evolution in the martensitic phase transformation. The transformation of B2–B19′ by the temperature-induced is simulated. The uniaxial tension and compression cases are performed to investigate the evolution of B19′ multi-variants and the mechanical response depends on SME and super-elasticity. Through the numerical simulations based on a phase field method, the following conclusions can be carried out in this paper.

(1) In the transition from the cubic B2 phase to the monoclinic B19′ phase with six martensite variants, the poly-twinned martensitic microstructure is formed by self-accommodating nucleation and growth. The evolution of the B19′ multivariants with different free-stress transformation strain causes the local deformation, resulting in the shape of the simulation area changing slightly.
(2) In the numerical simulation of the SME of NiTi SMA, the martensite detwinning takes place at the beginning of loading. The variants expand or vanish depending on the stress-free strain of B19′ multivariants and the direction of applied stress. The detwinning of the twinned martensitic structure finishes in a short time. After the detwinning, the material microstructure remains almost constant until the unloading is finished since the elastic energy of the material is minimal.
(3) The numerical simulation of the superelasticity behavior of the NiTi single crystal SMA accurately reproduces the complex evolution of microstructural morphologies during the martensitic and inverse phase transformation, and obtains the mechanical response of NiTi SMA in the process of loading-unloading.
(4) The results of the tension and compression test show an asymmetry for both the microstructure and mechanical response. The stress-accommodating martensite variants annex the ones of stress-unaccommodating gradually, and, thus, the different loading directions obtain different microstructures, which contain different martensite variants, resulting in various mechanical properties of NiTi SMA.

## Data Availability

The data presented in this study are available on request from the corresponding author.
